# Development of a Model of Care for Rehabilitation of People Living With HIV in a Semirural Setting in South Africa

**DOI:** 10.2196/resprot.3580

**Published:** 2014-12-04

**Authors:** Verusia Chetty, Jill Hanass-Hancock

**Affiliations:** ^1^University of KwaZulu-NatalDepartment of PhysiotherapySchool of Health SciencesDurbanSouth Africa; ^2^HEARDUniversity of KwaZulu-NatalDurbanSouth Africa

**Keywords:** model of care, rehabilitation, HIV, disability, physiotherapist, Delphi technique

## Abstract

**Background:**

Human immunodeficiency virus continues to challenge health care professionals even after the rollout of antiretroviral therapy. South Africa, among the worst affected countries in the world by the pandemic, has seen the effect of people living longer but facing disabling effects of both the virus and the associated impairments of the antiretroviral therapy. Rehabilitation within the evolving context of the disease has changed its focus from the impairment of the individual to the participation restriction within a person’s daily life. Offering a continuum of coordinated, multilevel, multidiscipline, evidence-based rehabilitation within health care will promote its prominence in health care structures.

**Objective:**

This study aims to develop a model of care within a health care structure using a semi-rural African setting as an example.

**Methods:**

The study will employ mixed methods using a Learning in Action Approach into the rehabilitation of people living with HIV (PLHIV) at the study setting. The Delphi technique, a multistage consensus method, will be used to obtain feedback from a number of local experts relevant for the field of rehabilitation of people living with HIV. The study will also involve various stakeholders such as the multidisciplinary health care team (doctors, physiotherapists, occupational therapists, dieticians, speech and language therapists, social workers, midlevel workers, community health care workers); department of health representative(s); site affiliated nongovernmental organization representative(s); and service users at the study setting.

**Results:**

Once a proposed model of care is derived, the model will be assessed for rigour and piloted at the study setting.

**Conclusions:**

The development of a model of care in rehabilitation for PLHIV in a health care setting is aimed to provide an example of a continuum of coordinated service throughout the disease trajectory. The assumption is that the burden on the health care system will be curbed and the projected benefit for all stakeholders will promote a sort after service delivery in rehabilitation of people living with HIV.

## Introduction

### Background

Human immunodeficiency virus (HIV) and the multiple effects on people living with the virus is an unequivocal health crisis facing the world at large. Sub-Saharan Africa remains the region worst affected [1]. South Africa is a middle income country at the tip of the continent that is most affected by the HIV epidemic [2]. The growing epidemic coupled with resource-poor settings in South Africa poses a challenge to the health care systems and their provision of a coordinated, multifaceted, and collaborated service from the national to the district level [2,3]. Rehabilitation is essential in facilitating patients’ functional independence and has a major role in the patient flow across the health care continuum. The disabling effects of HIV together with the associated disablements of antiretroviral therapy (ART) [4,5] demands that the team of health care professionals who are involved in rehabilitation offer a range of services in managing challenges encountered by people living with HIV through a comprehensive, coordinated, and collaborated program throughout the disease trajectory [6].

A model of care in rehabilitation has the prospect to reshape service delivery, patient outcomes, efficiencies, and collaboration with health care providers across the health system. A model of care is defined as:

A multifaceted concept, which broadly defines the way in which health care is delivered including the values and principles; the roles and structures; and the care management and referral processes. Where possible the elements of a model of care should be based on best practice evidence and defined standards and provide structure for the delivery of health services and a framework for subsequent evaluation of care. [7]

The impact of a model of care on the facilitative process between strategic directions for a health system to the delivery of care at local rehabilitation level is the goal within any strategic initiative [7].

The dearth of literature and lack of models of care to roll out rehabilitation for people living with HIV is astounding even though HIV and acquired immune deficiency syndrome (AIDS) are endemic in Southern Africa and therefore pose new issues to health and rehabilitation professionals in the region. Well-resourced countries have some approaches on the management of the disability in the context of HIV [8,9] However epidemic countries are still lacking such an approach and this does not only influence individual livelihoods related to disability adjusted life years but also adherence at larger scale. Thus rehabilitation needs to be integrated into the response to HIV and AIDS. The question is now not so much related to the “if” but “how” this can be done in resource poor settings such as South Africa. What model of care would address the rehabilitation needs of people living with HIV in the chosen semirural setting? Would the proposed model of care influence the rehabilitation practice at the above setting?

The aim of this study is to develop a model of care for people living with HIV in order to achieve the desired rehabilitation outcomes within a health care setting in the chosen semirural area. The objectives are to:

1. Review the existing literature and current models of care in rehabilitation;
2. Explore the perceptions of all stakeholders, that is, the multidisciplinary health care team (doctors, physiotherapists, occupational therapists, dieticians, speech and language therapists, social workers, midlevel workers, community health care workers), department of health representative(s), site affiliated nongovernmental organization (NGO) representative(s) and service users (people living with HIV receiving rehabilitation);
3. Identify criteria to be considered for the inclusion in the proposed model;
4. Develop the proposed model of care in collaboration with experts;
5. Appraise the methodological process in which the model was developed; and
6. Pilot the model of care for rehabilitation of people living with HIV within a semirural setting.

### Conceptual Framework

The research will be guided by an adapted version of the “Learning in Action” approach developed by the Athena Institute in Amsterdam [10]. The study will include several sub-studies which will logically flow into each other and be developed in cooperation with the nongovernmental community partner and the Department of Health.

### Approach

The process of innovation is a highly complex social set of activities, which can be conceived as an interactive, spiral process resulting from the interactions of a number of actors [10]. This implies that not only technical, but also social, organizational, political, economic, and cultural factors influence the development of innovations and the outcomes of social or technical change thereafter. The process of any type of research that focuses on innovation is therefore important to develop useful technology and interventions. However this process is often underestimated and not sufficiently understood.

On the basis of literature study and interviews the Athena Institute in Amsterdam developed the Interactive Learning in Action (ILA) approach. The approach is grounded in the following principles: active engagement of relevant stakeholders on equal footing early in the innovation process, explicit use of experiential knowledge, and knowledge creation through mutual learning (via dialogue), coalition building, and facilitated process with an emergent design. The overall process is a dynamic and cyclic process of activities, in which tentative results are tested and refined in practice in an interactive way. The ILA method comprises of five phases: (1) preparation and exploration; (2) in-depth study of problems, needs, and visions of each stakeholder group separately; (3) integration of different perspectives of stakeholder groups through mutual learning; (4) prioritization and agenda setting; and (5) implementation through reflexive cycles of planning, action, observation, reflection, and re-planning [10]. Essential for the successful execution of the approach is a well-filled tool box of methods and tools for different functions, which can be adapted to the local context and dynamics. A combined use of qualitative and quantitative methods enables the collection and comparison of a large diversity of perspectives, and is therefore preferred.

The phased activities structured along the lines of the transdisciplinary ILA research approach allows a process of integration. In practice, the process is cyclical and dialogical; for example, the output of one stakeholder group forms the input for another group, so that information gets extensive deliberation and rigorous redefinition to a point of being widely understood and acknowledged as relevant for practical use. [10]

Each substudy of this project will be integrated in the ILA approach and focus on different levels that can influence the development of a model of care in rehabilitation to address the disabling effects of HIV in a hyperepidemic country.

## Methods

### Overview

The study design is a mixed methods study combining both qualitative and quantitative methods. Qualitative methods will provide rich contextual information to inform the researcher on the subjective reality experienced by participants. The quantitative methods will be employed to seek consensus and rigour in decision making and implementation of the study using the Delphi technique. Adopting this approach will lead to the convergence of expert opinion in developing a model of care where none previously existed [11]. It will allow for facilitation from international evidence based and expert informed forum to a nationally guided and local level implementation of a model of care within a rehabilitation framework.

### Ethical Considerations

The study protocol received full ethical clearance from The University of KwaZulu-Natal, South Africa (HSS/1319/012D).

### Setting

The study setting is a 200-bed, level one district hospital, situated on the outskirts of the suburb of Durban, in the Mariannhill Mission Complex of the province of KwaZulu-Natal, South Africa. St Mary’s Hospital provides a service for 4500 people living with HIV. It is estimated that more than 250,000 people (33%) living in the St Mary’s Hospital catchment area are HIV-positive. Due to the dramatic increase in the numbers of patients suffering from AIDS-related illnesses, St Mary’s has focused its attention on improving and increasing its capacity to render holistic health care to HIV/AIDS patients and their families through various intervention programmes [4].

### Recruitment and Selection of Participants

Participants will include stakeholders relevant to a multidisciplinary health care team (doctors, physiotherapists, occupational therapists, dieticians, speech and language therapists, social workers, midlevel workers, community health care workers), department of health representative(s), site affiliated NGO representative(s) and service users (people living with HIV receiving rehabilitation). The Delphi technique will involve experts within the physiotherapy profession with experience in HIV and rehabilitation will comprise an additional part of the panel for this consensus technique in the consequent rounds.

### Data Analysis

The researcher will adopt the methodological approach of Van Manen by turning to the “nature of the lived experience, existential investigation, phenomenological reflection and conclude by phenomenological writing” [12], in analyzing the qualitative data. The Delphi multiple round questionnaires will be analyzed for level of agreement using Cronbach alpha for level of internal consistency against *a priori* limits. [13].

## Results

The study has been initiated by exploring the perceptions of all stakeholders, that is, the multidisciplinary health care team (doctors, physiotherapists, occupational therapists, dieticians, speech and language therapists, social workers, midlevel workers, community health care workers), department of health representative(s), site affiliated NGO representative(s) and service users (people living with HIV receiving rehabilitation). A focus group discussion was held at the study site and the results are currently being integrated into a Delphi questionnaire.

## Discussion

There is a paucity of studies in the literature addressing a model of care in the rehabilitation of people living with HIV. This study proposes the development and evaluation of a model of care using the ILA conceptual framework developed by the Athena institute which is a multilevel ILA framework incorporating a socioecological design focusing on an individual to policy development which will guide the structuring of the new “model of care in rehabilitation” within the study setting. A model of care for the rehabilitation of people living with HIV is novel and using sound theoretical discourse and evaluation will allow it to evolve from a single site study into advocating a national model to curb the burden on rehabilitation offered to people living with HIV. The study will draw on the convergence of expert opinion within the field of rehabilitation and hence provide sound theoretical judgement and critique in its development. It will allow researchers the platform to amalgamate and facilitate international evidence and expert opinion into a nationally guided and locally implemented model of care within a rehabilitation framework.

## Multimedia Appendix 1

Grant award letter.

## Abbreviations

AIDS: acquired immune deficiency syndrome
ART: antiretroviral therapy
HIV: human immunodeficiency virus
ILA: Interactive Learning in Action
NGO: nongovernmental organization
